# Differential Effect of Cytokines on CD4+ T‐Cell Activation, Metabolism, and Susceptibility to HIV‐1 Infection

**DOI:** 10.1155/jimr/7633359

**Published:** 2026-09-02

**Authors:** Amal Elfidha, Erick De la Torre Tarazona, Caroline Passaes, José Alcamí, Michaela Müller-Trutwin, Asier Sáez-Cirión

**Affiliations:** ^1^ Viral Reservoirs and Immune Control Unit, Institut Pasteur, Université Paris Cité, Paris, France, u-paris.fr; ^2^ Unité HIV Inflammation et Persistance, Institut Pasteur, Université Paris Cité, Paris, France, u-paris.fr; ^3^ Infectious Diseases Department, Hospital Ramón y Cajal, Instituto Ramón y Cajal de Investigación Sanitaria, Madrid, Spain, irycis.org; ^4^ AIDS and HIV Infection Group, Fundació de Recerca Clínic Barcelona-Institut d’Investigacions Biomèdiques August Pi i Sunyer (FRCB-IDIBAPS), Barcelona, Spain

**Keywords:** γc cytokines, CD4+ T-cells, cytokines, gamma-chain cytokines, HIV-1, IFN-α, IL-10, IL-16, immunometabolism, JAK/STAT, metabolism

## Abstract

HIV‐1 reservoirs are predominantly located in CD4+ T‐cells; however, not all CD4+ T‐cells contribute to the reservoir in the same way. Factors such as activation, differentiation, and cell metabolism have been proposed to determine the relative susceptibility of cells to HIV‐1 infection. HIV‐1 reservoirs are seeded early during the acute phase of infection, but the cell composition of these reservoirs evolves in the transition to chronic infection. This suggests that there are factors during the acute phase that may alter the intrinsic susceptibility of CD4+ T‐cells to HIV‐1. We investigated here the influence of common cytokines known to be secreted during the acute phase and to play a role in either T cell homeostasis or HIV‐1 infection on activation, differentiation, metabolic activity, and HIV‐1 susceptibility of CD4+ T‐cells. We show that the proinflammatory cytokines interleukin (IL)‐2, IL‐7, and IL‐15 induce cellular activation, differentiation to effector profile, increase in metabolic capacity, and HIV‐1 susceptibility. In contrast, IL‐21, another cytokine from the gamma‐chain (γc) family, showed opposing effects on the same parameters, including decreasing oxidative phosphorylation (OXPHOS) and HIV‐1 infection. We also show that IL‐10 and interferon (IFN)‐α are able to at least partially revert the cellular and metabolic changes induced by IL‐2 or IL‐7 and reduce the cells’ susceptibility to HIV‐1.

## 1. Introduction

Despite the success of antiretroviral therapy (ART) in reducing HIV‐1 replication and in preventing or reversing immunodeficiency in people living with HIV (PLWH), the persistence of an HIV reservoir is a roadblock to an HIV cure. As such, integrated replication‐competent proviruses in the reservoir cells drive a rapid viral rebound within weeks of ART cessation. This persistent reservoir is the focus of extensive research efforts. There are various types of HIV‐harboring cells; however, most of the HIV reservoir consists of CD4+ T lymphocytes. Nevertheless, CD4+ T lymphocytes are heterogeneous, and the contribution of different CD4+ T‐cell subsets to the reservoir varies [1, 2]. In this regard, memory CD4+ T subsets, such as stem‐like memory cells (TSCM), central (TCM), and transitional (TTM) memory CD4+ T‐cells, have been shown to be enriched in HIV proviral DNA [1, 2]. The heightened susceptibility to HIV infection, the survival and self‐renewal capacities, and the inherent extended life span of these memory subsets provide an optimal environment for viral persistence.

The formation of the HIV reservoir occurs within the first days following infection; however, the composition of the reservoir appears to vary throughout infection. Early ART initiation has been associated with a significant reduction in the size of the reservoir and a delay in viremia rebound upon treatment cessation [3–7]. Furthermore, early ART initiation was reported to reduce the contribution of cells with self‐renewal characteristics, such as TSCM and TCM cells, to the overall HIV reservoir, a trait also observed in persons who control HIV without ART [3, 8–12]. These observations suggest that the early viral reservoir may be more labile [13], and the susceptibility of CD4+ T‐cells to HIV‐1 infection is altered during primary infection. Environmental factors such as cytokines released during AHI [14], which modulate the activation, differentiation, and function of CD4+ T‐cells, may influence their intrinsic permissibility to HIV‐1 infection [14–16].

Changes promoting CD4+ T‐cell response and differentiation provide an adequate environment for HIV‐1 replication since HIV‐1 infection susceptibility varies with T‐cell differentiation, with effector memory cells being highly permissive and naive T cells being significantly resistant to infection [17–19]. Similarly, following activation by a cognate foreign antigen or cytokines, CD4+ T lymphocytes become more permissive to HIV‐1 infection, and a multitude of studies showed that HIV infection of activated CD4+ T‐cells is much more efficient than that of resting cells [20–22]. In fact, CD4+ T‐cells exhibit fast clonal proliferation upon stimulation, and it is well known that the intracellular environment linked to cell cycle entry favors HIV‐1 infection. The chromatin environment of the integrated provirus and the transcriptional state of the CD4+ T‐cell subset that carries latent HIV‐1 have both been attributed to this unique intracellular milieu.

We have shown that HIV‐1 preferentially infects CD4+ T‐cells with high metabolic activity [23]. We proposed that the increased permissibility of CD4+ T‐cells is controlled by modifications to the cellular metabolism, which drive the differentiation process. Indeed, metabolism is a key regulator of the biology of T cells. Engagement of the T cell receptor (TCR) and costimulatory signals sets off a metabolic switch that promotes protein, fatty acid, and nucleotide production and biomass accumulation to promote cell proliferation and effector activities [24].

Several studies have explored the involvement of cytokines, chemokines, and antigenic signals in shaping CD4+ T‐cell activation and promoting HIV‐1 infection. We explored here whether some of these cytokines may act by altering the default homeostatic CD4+ T‐cell metabolic profile. We focused on cytokines known to be secreted early during acute infection, to have an impact on HIV‐1 replication, and to play a significant role in CD4+ T‐cell homeostasis. We studied specifically the effects of interleukin (IL)‐2, IL‐7, IL‐10, IL‐15, IL‐16, IL‐21, and interferon (IFN)‐α2a (referred to as IFN‐α in this article). The common gamma‐chain (γc) cytokines IL‐2, IL‐7, IL‐15, and IL‐21 are involved in T cell homeostasis, development, expansion, and differentiation [25]. IL‐21 is associated with Tfh and Th17 differentiation, two of the preferential targets of HIV infection [26, 27]. IL‐10 is a regulatory cytokine that has been shown to drive Treg cell responses in HIV infection and is associated with viral persistence on ART [28]. IL‐7 and the proinflammatory cytokines IL‐2 and IL‐15 have been rather associated with increased HIV‐1 replication [29, 30], whereas the anti‐inflammatory IL‐10 and IFN‐α2a were shown to inhibit HIV‐1 replication [31–33]. IL‐16 shares the main HIV‐1 receptor, CD4, and has been described to inhibit viral replication [34]. We investigated in vitro the impact of these immunomodulators on CD4+ T‐cell activation, differentiation, metabolism, and susceptibility to HIV‐1 infection.

## 2. Results

### 2.1. Divergent Effect of Cytokines on CD4+ T‐Cell Activation and Differentiation

**Figure 1:**
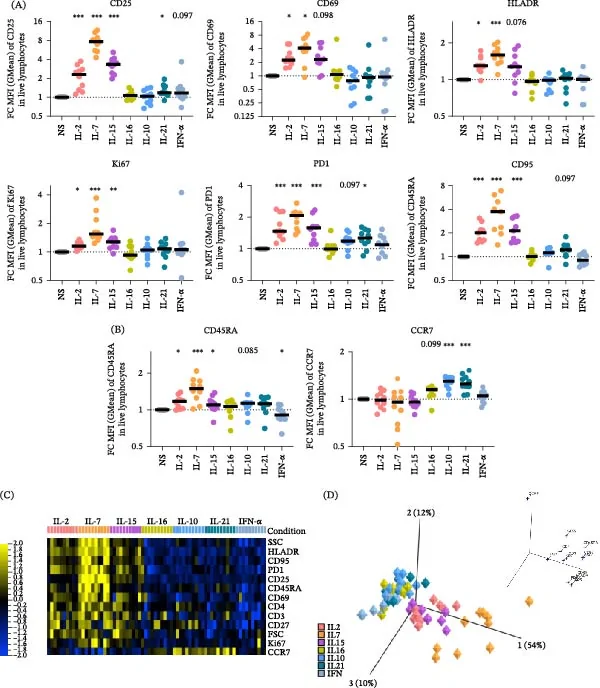
IL‐2, IL‐7, and IL‐15 increase cellular activation and differentiation of CD4+ T‐cells. Relative changes in the expression levels of CD25, CD69, HLADR, Ki67, PD1, and CD95 (A) and CD45RA and CCR7 (B) after 5 days of culture in the presence of the cytokines when compared to the control condition. Each dot corresponds to one sample. Lines are medians. (C) Heatmap representing the fold‐change in all the markers analyzed in our study. (D) Plot showing the principal component analyses comparing the different culture conditions (each symbol represents CD4+ T‐cells from one individual) and the contribution of the variables analyzed.

We first assessed the impact of IL‐2, IL‐7, IL‐10, IL‐15, IL‐16, IL‐21, and IFN‐α on the expression of several markers of activation and differentiation on purified CD4+ T‐cells from blood donors. As expected, IL‐2, IL‐7, and IL‐15 strongly activated CD4+ T‐cells, as shown by the upregulation of CD25, CD69, HLA‐DR, PD‐1, and CD95 after 5 days of culture compared to the nonstimulated condition (Figure 1A, Supporting Information 1: Figure S1). In addition, these cytokines induced robust proliferation, as indicated by the increased expression of the proliferation marker Ki‐67. In contrast, only a small increase in CD25 and PD‐1 was induced by IL‐21, and no significant effect on CD4+ T‐cell activation was observed for IL‐10, IL‐16, or IFN‐α when compared to the control condition (Figure 1A, Supporting Information 1: Figure S1). The changes in the activation markers induced by the different cytokines were accompanied by equivalent changes in CD4 expression levels and in the size and granularity of the cells (Supporting Information 1: Figure S1).

Accordingly, IL‐2, IL‐7, and IL‐15 promoted T cell differentiation (Figure 1B), which was evidenced by a decrease in the proportion of CD4+ T‐cells with a CM and EM profile and an increase of cells with an effector profile (TEMRA) (Supporting Information 1: Figure S2). In contrast, IL‐10 and IL‐21 increased the levels of CCR7 (Figure 1B) and decreased the proportion of cells with TM and TEMRA profiles when compared with the nonstimulated condition. A decrease in the proportion of TEMRA CD4+ T‐cells was also observed in the presence of IFN‐α. Overall, in our experimental conditions, IL‐7 was the cytokine that most efficiently stimulated CD4+ T‐cells, followed by IL‐2 and IL‐15, which had equivalent effects (Figure 1C,D). This was likely related to the higher constitutive presence of IL7R/CD127 among CD4+ T‐cells (Supporting Information 1: Figure S3). IL‐10, IL‐21, and IFN‐α did not have much effect on T cell activation but restrained T cell differentiation, while IL‐16 had a more neutral effect (Supporting Information 1: Figure S2).

### 2.2. Distinct Metabolic Signatures of Cytokines in CD4 T Cells

**Figure 2:**
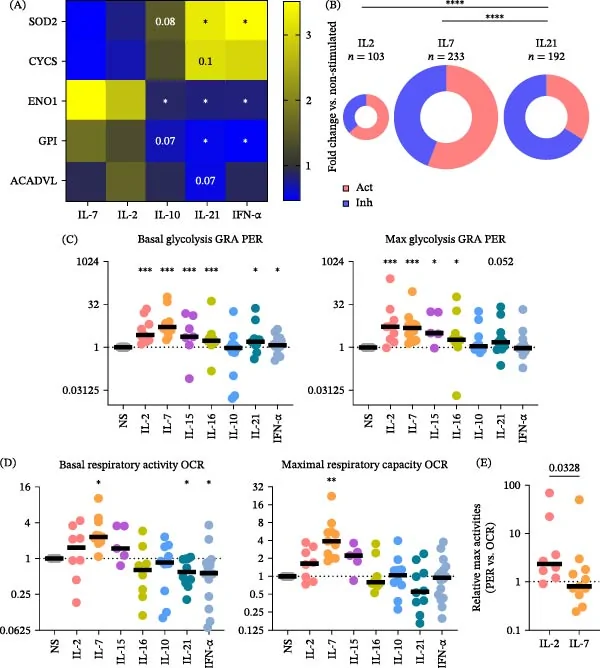
Contrasting effects of IL‐2, IL‐7, and IL‐15 vs. IL‐21 on the metabolic activity of CD4+ T‐cells. (A) Heatmap displaying genes that were differently expressed (kruskal–Wallis test) when compared to IL‐7 in response to IL‐2, IL‐10, IL‐21, or IFN‐α. Each column represents the geometric mean of results for CD4+ T‐cells from three individuals. (B) Differences in the proportion of upregulated (FC ≥ 1.5) vs. downregulated genes (FC < 0.67) in murine naïve CD4 T cells after stimulation with IL‐2, IL‐7, and IL‐21 vs. nonstimulated condition. Data were provided by Dr. Christophe Benoist [35]. The size of the chart is proportional to the number of regulated genes for each condition. Relative changes in basal and maximal glycolysis (C) and basal and maximal respiratory capacity (OXPHOS) (D) after 5 days of culture in the presence of the cytokines when compared to the control condition. (E) Ratio of maximal glycolysis and maximal OXPHOS in IL‐2 and IL‐7 conditions. Each dot corresponds to one sample and lines are medians.  ^∗^ *p*  < 0.05,  ^∗∗^ *p*  < 0.01,  ^∗∗∗^ *p*  < 0.001, and  ^∗∗∗∗^ *p*  < 0.0001.

To examine the impact of cytokines on the metabolic program of CD4+ T‐cells, we first analyzed the expression of a panel of genes related to the regulation of different metabolic pathways (Supporting Information 2: Table S1) in CD4+ T‐cells from three donors upon stimulation with IL‐2, IL‐7, IL‐10, IL‐21, or IFN‐α. A marked divergence was observed in the transcriptional profiles induced by the different cytokines (Figure 2A). IL‑7, similar to IL‑2, maintained elevated expression of glycolytic genes (ENO1, GPI), consistent with an active metabolic program. IL‑10, IL‑21, and IFN‑α stimulation, however, resulted in reduced expression of these metabolic genes and higher levels of genes associated with mitochondrial integrity and ROS management (CYC2, SOD2).

To gain transcriptional insight into the metabolic programs induced by γc cytokines, we analyzed publicly available scRNA‐seq data from murine naïve CD4+ T‐cells stimulated with IL‐2, IL‐7, or IL‐21 [35]. This dataset was selected because it provides a side‐by‐side comparison of these three γc cytokines in a homogeneous T‐cell population under standardized experimental conditions, enabling pathway‐level analysis of cytokine‐specific transcriptional responses. At the pathway level, IL‐7 emerged as a broad metabolic activator, inducing a large transcriptional program (*n* = 130 of 385 filtered genes with fold change [FC] ≥ 1.5) (Figures 2B and Supporting Information 1: Figure S4). In comparison, IL‐2 elicited a more restricted program (65 genes, with a substantial proportion—60% shared with the IL‐7‐induced gene set). In contrast, IL‐21 diverged markedly from this activating pattern. Although it induced a similarly limited number of genes (*n* = 66), its overall signature was predominantly repressive, with 127 downregulated genes (FC < 0.67). IL‐2 and IL‐7 downregulated a lower number of genes (*n* = 38 and *n* = 103 genes with FC < 0.67, respectively) and had an overall activating profile (Figure 2B). Overall, these data are consistent with our analysis of human CD4+ T‐cells, and, together, these results indicate that γc cytokines elicit partially overlapping yet functionally distinct transcriptional programs, which highlight the intertwined roles of the cytokines in regulating diverse metabolic pathways.

### 2.3. Different Impact of Cytokines on Glycolysis and Oxidative Phosphorylation (OXPHOS)

In order to determine the overall result of cytokine signaling on the metabolic activity of CD4+ T‐cells, we used a cell flux analyzer to measure the oxygen consumption rate (OCR) and the proton efflux rate (PER) as indicators of OXPHOS and glycolysis in the cells. IL‐7, IL‐2, and IL‐15 strongly increased the basal glycolytic activity of the cells and their maximal glycolytic capacity (Figure 2B). IL‐16 and IL‐21 slightly increased the glycolytic activities of CD4+ T‐cells. IL‐7 also strongly enhanced the basal respiratory activity and the maximal respiratory capacity of CD4+ T‐cells (Figure 2C). Enhancing effects were observed for IL‐2 and IL‐15 in most but not all samples analyzed (Figure 2C). We observed that while IL‐7 equally enhanced the maximal respiratory and glycolytic capacity of the cells, IL‐2 appeared to preferentially enhance the maximal glycolytic capacity (Figure 2D), which reached similar levels in cells cultured in the presence of IL‐2 and IL‐7 (Figure 2B). In contrast, IL‐21 and IFN‐α decreased the basal respiratory capacity of CD4+ T‐cells (Figure 2C). A reduction in the maximal respiratory capacity was also observed in cells cultured in the presence of IL‐21 when directly compared to the control condition (*p* = 0.04), but statistical significance was lost in the multiple comparison analysis (Figure 2C). Overall, these results confirm divergent effects on CD4+ T‐cell metabolism of IL‐21 and the other γc cytokines analyzed.

### 2.4. Contrasting Effect of Cytokines on the Susceptibility of CD4+ T‐Cells to HIV‐1 Infection

**Figure 3:**
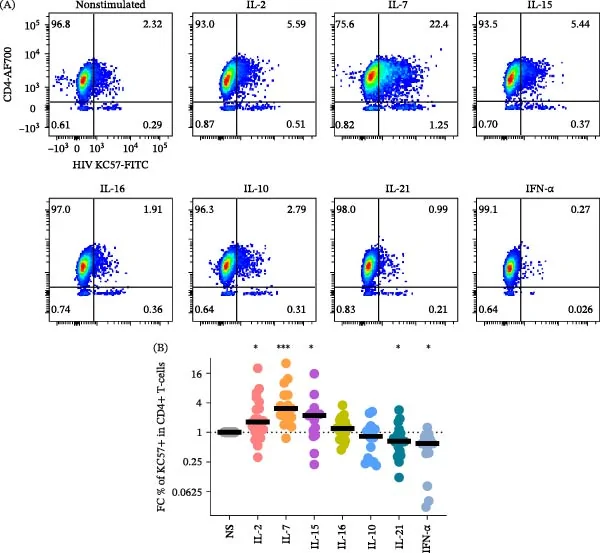
IL‐2, IL‐7, and IL‐15 enhanced, while IL‐21 and IFN‐α decreased HIV‐1 infection of CD4+ T‐cells. (A) Representative example of the frequency of HIV‐1 infection observed in the different culture conditions for CD4+ T‐cells from one donor. (B) Relative frequency of infected cells treated with different cytokines vs. the nontreated condition. Each dot corresponds to one sample. Lines are medians.

HIV‐1 infection has been proposed to increase with the activation status and metabolic activities of CD4+ T‐cells. We therefore analyzed the effect of the cytokines studied here on the susceptibility of CD4+ T‐cells to infection with replication‐competent HIV‐1 BaL. We found that IL‐2, IL‐15, and particularly IL‐7 enhanced the frequency of HIV‐1‐infected cells when compared to the nonstimulated condition (Figure 3). In contrast, the number of infected cells was reduced when the cells were cultured in the presence of IL‐21 or IFN‐α, resembling the effects that were observed for basal mitochondrial activity. No significant effects were observed for IL‐10 or IL‐16 under these conditions.

### 2.5. IL‐10 Antagonized the Enhancing Effects of IL‐2 and IL‐7 on CD4+ T‐Cell Metabolic Activity and Susceptibility to HIV‐1 Infection

**Figure 4:**
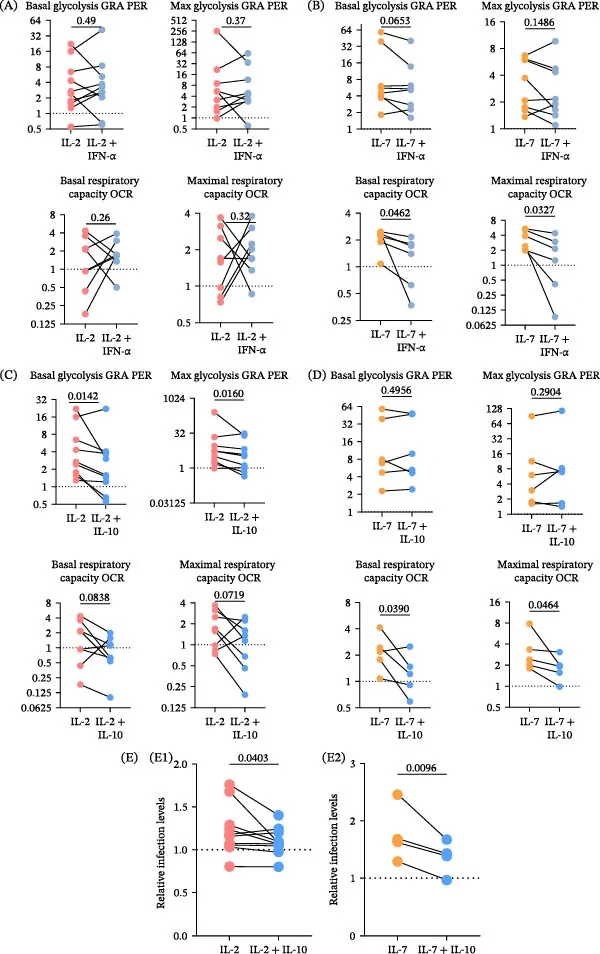
Impact of IL‐10 and IFN‐α on counteracting the metabolic enhancing effects of IL‐2 and IL‐7. Relative metabolic activities (vs. nontreated condition) in cells treated with IL‐2 in the absence or presence of IFN‐α (A) or IL‐10 (C). Relative metabolic activities (vs. nontreated condition) in cells treated with IL‐7 in the absence or presence of IFN‐α (B) or IL‐10 (D). (E) Relative infection levels of cells treated with IL‐2 (E1) and IL‐7 (E2) in the absence or presence of IL‐10. Each dot corresponds to one sample. Lines are medians.

We wondered whether the potentially repressing effect of some cytokines, such as IFN‐α or IL‐10, might have been masked by the relative quiescence of the CD4+ T‐cells that we used ex vivo. We therefore explored whether these cytokines could counteract the enhancing effects that were observed when cells were cultured in the presence of IL‐2 and/or IL‐7. We found that whereas the absence or presence of IFN‐α did not alter the metabolic activities of CD4+ T‐cells cultured with IL‐2 (Figure 4A), the additional presence of IFN‐α dampened the metabolic activities, notably the respiratory capacity, of the cells that were cultured in the presence of IL‐7 (Figure 4B).

Conversely, cells cultured in the presence of both IL‐2 and IL‐10 showed lower glycolytic activity and potential than cells cultured in the presence of IL‐2 alone, and a trend was also observed towards a decrease in OXPHOS in the presence of IL‐2+IL‐10 vs. IL‐2 alone (Figure 4C). The presence of IL‐10 did not alter the glycolytic capacity of cells that were cultured in the presence of IL‐7 but did decrease their basal respiratory activity and maximal respiratory capacity (Figure 4D). Moreover, the presence of IL‐10 decreased the frequency of HIV‐1 infected cells that were observed when CD4+ T‐cells were cultured with IL‐2 or IL‐7 alone (Figure 4E). These results indicate that cytokines may show different antagonizing effects on CD4+ T‐cell metabolic activities. While IL‐2 appears more sensitive to the antagonizing effects of IL‐10 than to IFN‐α, the mitochondrial activities enhanced by IL‐7 are reduced in the presence of both IL‐10 and IFN‐α.

## 3. Discussion

In this study, we compared the effects of selected cytokines on CD4+ T‐cell activation, differentiation, metabolism, and susceptibility to HIV‐1 infection in vitro. We found that some common γc cytokines, particularly IL‐7, strongly enhanced CD4+ T‐cell activation, promoted differentiation toward effector phenotypes, and increased metabolic activity, associations that correlated with heightened susceptibility to HIV‐1 infection. In contrast, IL‐21 and IFN‐α exhibited inhibitory profiles, dampening both metabolic activation and HIV‐1 replication relative to IL‐2 or IL‐7 cytokines. Notably, IL‐10 demonstrated a unique immunoregulatory function, counteracting the pro‐infectious effects of IL‐2 and IL‐7. This antagonism was accompanied by selective dampening of OXPHOS in response to IL‐7 and glycolysis in response to IL‐2. Together, these findings highlight the diverse and sometimes opposing roles of cytokines in shaping CD4+ T‐cell immunometabolic states and underscore how such modulation can critically influence HIV‐1 susceptibility.

The common γ‐chain cytokines, IL‐2, IL‐7, and IL‐15, which play essential roles in peripheral T cell growth and survival, exhibited, as expected, comparable enhancing effects on all markers of cellular activation. This increase in cell activation was accompanied by a significant decline in both the T_CM_ and T_EM_ subsets while increasing the T_EMRA_ subset. These observations are consistent with previous studies indicating that, in the absence of antigenic stimulation, T_CM_ cells can differentiate into CD45RA+CCR7‐ T_EMRA_ cells in the presence of IL‐2, IL‐7, and IL‐15, emphasizing the importance of these cytokines as factors in the development and maintenance of the functional effector CD4+ T‐cell response [36, 37]. IL‐2, IL‐7, and IL‐15 all signal through a shared receptor γ‐chain and a common STAT5‐dependent pathway [25], which likely underpins their overlapping effects.

In our study, IL‐7 showed the strongest effect when compared to IL‐2 and IL‐15, which might be explained in part by the higher constitutive expression of IL‐7Rα (CD127) by CD4+ T‐cells in the absence of stimulation [38], compared to IL‐2Rβ (CD122), a receptor component shared by IL‐2 and IL‐15 [39–41]. The relatively modest effect of IL‐15 in our experiments may relate to its dependence on trans‐presentation via IL‐15Rα for high‐affinity signaling. IL‐15Rα is not substantially expressed by resting CD4+ T‐cells but is predominantly found on antigen‐presenting cells [41, 42]. Despite these differences, all three cytokines significantly increased CD4+ T‐cell susceptibility to HIV‐1 infection, in line with prior studies [43].

Despite their shared capacity to activate CD4+ T‐cells, these γc cytokines exhibit subtle yet functionally significant differences in their stimulatory profiles. This divergence was most evident in the metabolic readouts. IL‐7 induced a coordinated upregulation of both glycolysis and OXPHOS, indicating balanced metabolic activation. In contrast, IL‐2 and IL‐15 primarily increased glycolytic activity, with comparatively modest effects on mitochondrial respiration. Notably, IL‐2 significantly boosted the maximal glycolytic capacity to levels approaching those seen with IL‐7, suggesting an IL‐2 metabolism skewed toward glycolysis. These findings highlight nuanced functional differences among γc cytokines, particularly in how they modulate T‐cell bioenergetics. Previous studies have shown that IL‐2, IL‐15, and, to a lesser extent, IL‐7 stimulate mTORC1 activation in CD4+ T‐cells [44]. mTORC1 is known to stimulate glycolysis, which may explain why the relative increase in glycolysis in CD4+ T‐cells treated with IL‐2 was higher than that in IL‐7. Conversely, IL‐7 has been reported to enhance glycerol uptake, facilitating triglyceride synthesis and storage, thereby supplying substrates for FAO [45]. This implies that IL‐7 mobilizes FAO as well as glycolysis to fuel the TCA cycle, explaining the high increase in OXPHOS [46]. These metabolic profiles are in line with the transcriptomic analyses of cytokine‐stimulated murine naïve CD4+ T‐cells, which revealed a broad metabolic activation signature induced by IL‐7 and a more restricted pattern focused on carbohydrate metabolism in response to IL‐2, despite a substantial overlap in the genes regulated by both cytokines.

IL‐21, another γc cytokine, exhibited markedly distinct effects compared with those of IL‐2, IL‐7, and IL‐15. While a shared feature of γc cytokines is the upregulation of PD‐1 on purified T cells [47], IL‐21 treatment in our study induced PD‐1 and CD25 expression but did not significantly alter the levels of other activation markers such as CD69, HLA‐DR, CD95, or the proliferation marker Ki67. This is in line with prior observations that IL‐21 can enhance CD25 expression and sensitize T cells to IL‐2 signaling [48, 49]. However, its capacity to induce broader T cell activation markers appears limited and highly context‐dependent, requiring costimulation or synergistic cytokine signaling to fully activate effector responses [50–52].

Although IL‐21 shares the common γc receptor subunit with other members of the family, it primarily signals through STAT3, rather than STAT5, which is predominantly activated by IL‐2, IL‐7, and IL‐15 [53]. This distinct signaling pathway may explain why IL‐21’s functional effects resemble those of IL‐10, another cytokine that signals mainly through STAT3 [54, 55]. IL‐10 did not show any discernible effect on CD4+ T‐cell activation in our conditions, but both IL‐10 and IL‐21 preserved the frequency of the memory subsets, maintaining CCR7 expression, and decreasing the contribution of the more differentiated T_TM_, T_EM_, and T_EMRA_ to the pool of CD4+ T‐cells. Consistent with these findings, IL‐21 has been shown to promote the generation of stem cell‐like memory T cells (TSCM) following short anti‐CD3/CD28 stimulation [56]. These properties have attracted considerable interest in adoptive cell therapy, where IL‐21 is increasingly used to preserve early memory characteristics, enhance persistence, and improve the quality of therapeutic T‐cell products [57]. Although IL‐21 caused a very modest increase in basal and maximal glycolysis capacity, it led to a significant reduction in OXPHOS. This shows that IL‐21 regulates these major metabolic pathways differently from IL‐2, IL‐7, and IL‐15. Contrasting effects on the metabolic activity of IL‐2 and IL‐21 have already been reported for CD8+ T cells [58]. The transcriptomic analyses of murine CD4+ T‐cells further underlined an overall inhibitory metabolic profile induced by IL‐21, which affected genes involved in both carbohydrate and lipid metabolism. IL‐21 has been shown to induce IL‐10 expression in various immune cells, including CD4+ T‐cells and B cells [50, 52], which could contribute to its immunoregulatory and partly inhibitory profiles, paralleling IL‐10’s effects. This IL‐21–IL‐10 axis may mediate feedback control, limiting excessive T cell activation and inflammation [59]. While other γc cytokines such as IL‐2 and IL‐15 have also been reported to induce IL‐10, this effect is typically more conditional and context‐dependent. In contrast, IL‐21 more robustly promotes IL‐10 expression in several T‐cell subsets and is uniquely positioned, via STAT3 signaling, to support a regulatory transcriptional program resembling that of IL‐10 itself. We could not observe any impact of IL‐10 on the metabolic activity of CD4+ T‐cells when added to nonstimulated cells. In contrast, IL‐10 inhibited the metabolic enhancement caused by IL‐2 and IL‐7, consistent with its known anti‐inflammatory rather than activating role. Interestingly, IL‐10 selectively inhibited the dominant metabolic pathway induced by each cytokine: it counteracted the IL‐2‐driven increase in glycolysis without significantly affecting OXPHOS and suppressed IL‐7‐induced mitochondrial respiration with no effect on glycolysis. IL‐10 has been shown to counteract mTORC1‐induced glycolysis and may have this action in IL‐2‐treated cells [60]. This differential inhibition reinforces the notion that IL‐2 and IL‐7 elicit distinct metabolic programs in T cells and highlights IL‐10’s ability to fine‐tune specific cytokine‐induced metabolic outputs rather than broadly suppressing activation.

We found that IFN‐α, a type I cytokine known to modulate T‐cell responses through direct signaling via IFNAR on T cells [61, 62], did not provoke major changes in T‐cell activation and differentiation, except decreasing the proportion of effector cells and CD4 and CD27 levels in most of the samples analyzed. IFN‐α did not alter the glycolytic activities of CD4+ T‐cells but decreased their basal respiratory activity. Moreover, IFN‐α did not counteract the metabolic changes induced by IL‐2 (biased towards glycolysis) but decreased the metabolic activities induced by IL‐7, in particular the basal and maximal respiratory capacities. These results suggest that IL‐2 is relatively resistant to IFN‐α’s suppressive effects and that IFN‐α is involved in the regulation of OXPHOS in CD4+ T‐cells.

The impact of type I IFNs on immune cell metabolism is increasingly recognized but remains complex and context‐dependent [63]. While several studies report that IFN‐α can promote mitochondrial biogenesis and enhance OXPHOS in certain cell types, such as plasmacytoid dendritic cells and nonhematopoietic cells [64], evidence indicates that in T cells, particularly under chronic exposure or in disease contexts, IFN‐I can impair mitochondrial function [65]. In multiple sclerosis patients treated with IFN‐β, a dose‐dependent reduction in intracellular ATP levels and mitochondrial membrane potential was observed in CD4+ T‐cells, indicating impaired mitochondrial activity [66]. Similarly, in systemic lupus erythematosus, CD8+ T cells exposed to prolonged IFN‐α stimulation exhibited decreased mitochondrial gene expression and reduced spare respiratory capacity, suggesting compromised mitochondrial function [67]. Moreover, IFN‐α has been reported to inhibit IL‐7‐induced proliferation of CD4+ T‐cells by selectively impairing Akt phosphorylation, a key signaling event downstream of IL‐7 receptor engagement, while not affecting STAT5 phosphorylation [62]. In the tumor microenvironment, chronic IFN‐I signaling has been shown to drive terminal exhaustion of CD8+ T cells by inducing lipid peroxidation and metabolic dysfunction, thereby limiting their antitumor efficacy [68].

The capacity of IL‐10, IL‐21, and IFN‐α to downmodulate the metabolic activities of CD4+ T‐cells, measured using extracellular flux analysis, was accompanied by a capacity of these cytokines to reduce the susceptibility of the cells to HIV‐1 infection, either directly or by dampening the enhancing effects of IL‐2/IL‐7. Although we have not demonstrated a direct association between these effects, we and others have previously shown that the specific inhibition of metabolic activities in CD4+ T‐cells blocks HIV‐1 infection [23, 69]. It is therefore tempting to propose that the capacity of the cytokines evaluated here to enhance or repress cell metabolism may contribute to shaping their susceptibility to HIV‐1 infection. Moreover, our results suggest that the capacity of IFN‐α to rewire the CD4+ T‐cell metabolic program could reinforce the refractory activities of IFN‐induced antiviral factors. Our results indicate that the “cytokine storm” that occurs during acute HIV‐1 infection may decisively influence the fate of CD4+ T‐cells in terms of HIV‐1 infection and contribute to explaining why HIV susceptibility varies dynamically across different cell types and infection stages.

Our study is limited to a single concentration of ILs on bulk primary CD4+ T‐cells, and we cannot exclude that their effects could differ at other doses and in a more dynamic complex model [70, 71]. Additionally, variable expression levels of γc receptors and associated subunits among CD4+ T‐cell subsets could influence sensitivity and responsiveness to these cytokines [72], and future studies using subset‐resolved or single‐cell approaches will be necessary to define how naïve, memory, regulatory, and effector T‐cell populations differentially contribute to the metabolic and functional programs described here. Nevertheless, our analysis reveals that while γc cytokines share the common γc receptor component and engage common JAK signaling components, each cytokine triggers a distinct transcriptional signature that differently influences metabolic gene regulation. This nuanced interplay helps explain why these cytokines can have such cell type‐ and context‐specific effects, as recently shown by Baysoy et al. [35]. Extending the analysis to non‐γc cytokines suggests that differences in STAT utilization may contribute to the emergence of distinct metabolic programs. An additional limitation of this study is that the transcriptional data analyzed were derived from murine naïve CD4+ T‐cells and therefore may not fully recapitulate human T‐cell gene regulation. Nevertheless, the major signaling pathways engaged by γc cytokines, including JAK/STAT and PI3K‐AKT‐mTOR signaling, are highly conserved across species, and both the murine transcriptomic data and human functional assays identified divergent responses to IL‐2, IL‐7, and IL‐21, strengthening the conclusion that these cytokines differentially regulate T‐cell metabolic programs.

Our results provide a comparative overview of the complex effects that cytokines have on T‐cell activation, differentiation, and cell metabolism and their possible impact on their susceptibility to HIV‐1 infection. Although it is generally considered that metabolism, activation, and differentiation are intertwined processes, our study shows that, although generally interrelated, they are not immediately equivalent and are subtly regulated, leading to differences that may affect the sensitivity of the cells to complementary or antagonistic signaling and their susceptibility to HIV‐1 infection. The translational implications of these findings should be interpreted with caution. Although cytokines such as IL‐2 and IL‐7 have shown promising immunostimulatory activity in preclinical studies, clinical trials evaluating systemic cytokine administration in HIV infection and cancer have generally produced limited or inconsistent benefits. These outcomes underscore the context‐dependent nature of cytokine biology, where the cellular state, differentiation stage, tissue environment, and integration with other signaling pathways collectively determine biological responses. Nevertheless, cytokines remain critical tools as adjuvants and in adoptive cell therapy manufacturing, where they are used to program cellular differentiation, persistence, and functionality. Our findings may therefore help guide the optimization of next‐generation T‐cell therapies, particularly as metabolic programming and engineering emerge as major strategies to enhance therapeutic efficacy.

## 4. Methods

### 4.1. Isolation and Culture of Primary Human CD4+ T‐Cells

All experiments were conducted using primary human cells. Blood samples from voluntary donors were obtained from the French blood bank (Etablissement Français du Sang) as part of an agreement with the Institut Pasteur (C CPSL UNT, number 15/EFS/023).

Peripheral blood mononuclear cells (PBMCs) were isolated from donor‐derived buffy coats. Buffy coats were diluted 1:1 (vol/vol) in a phosphate‐buffered saline (PBS) buffer, overlaid on a density gradient medium, Lymphocyte Separation Medium (EuroBio), at a ratio of 2:1 blood to Ficoll, and then centrifuged at 1800 rpm for 30 min at minimum acceleration/deceleration to harvest PBMCs. Red blood cells (RBCs) were removed using 1× RBC lysis buffer.

CD4+ T‐cells were then purified from the PBMCs by negative selection using the Stemcell EasySep Human CD4+ T‐cell enrichment kit. CD4+ T‐cell‐enriched PBMCs show purities greater than 90%. Cells were cultured at a density of 10^6^ cells/mL in RPMI 1640 containing Glutamax (ThermoFisher), supplemented with 10% fetal calf serum (FCS), penicillin (10 IU/mL), and streptomycin (10 μg/mL) (referred to below as culture medium). Cells were then maintained at 37°C in a standard tissue culture humidified incubator containing atmospheric (20%) O_2_ and 5% CO_2_.

### 4.2. Treatment of CD4+ T‐Cells With ILs

Depending on the condition, CD4+ T‐enriched PBMCs were either left nonactivated in culture medium (nonstimulated condition, NA) or stimulated by adding either recombinant human (rh) IL‐7, rhIL‐10, rhIL‐15, rhIL‐16, rhIL‐21 (all at 10 ng unless indicated otherwise, from R&D Systems) or rhIFN‐α2b (at 100 U, Sigma) to each well. The cells were then maintained in culture for 5 days prior to infection or analysis, as previously described [23].

### 4.3. Infection of Primary CD4+ T‐Cells

Activated CD4+ T‐cells were infected with HIV‐1 BaL (R5) (7 ng/mL p24) virus by centrifuging at 1200 × g for 1 h at room temperature and then incubating for 1 h at 37°C in a humidified 5% CO_2_ incubator [73]. The cells were then washed once with PBS and incubated in the culture medium. Infection levels were evaluated on day 3 postinfection using flow cytometry, by HIV‐1‐Gag protein and p24 intracellular staining (p24‐FITC) (clone KC57, Coulter) [74].

### 4.4. Activation Phenotyping

Cells were first stained with the LIVE/DEAD Fixable Aqua Dead Cell Stain Kit, for 15 min at room temperature, then washed then washed in PBS plus 1% FCS prior to staining with activation markers. Cell activation was monitored using fluorochrome‐conjugated monoclonal antibodies (mAbs) (from BD Biosciences, Beckman Coulter, Biolegend, ThermoFisher, or Milteyni). We used the following antibodies: CD3‐eFluor450 (clone SK7), CD4‐AlexaFluor700 (OKT4), HLA‐DR‐FITC (G46‐6), CD25‐ECD (B1.49.9), CD69‐PE‐Cy7 (FN50), and PD‐1‐PE (eBioJ105).

CD4+ T‐cells were incubated with the surface marker antibodies for 20 min at 4°C and then washed twice in PBS plus 1% FCS. For the intracellular staining, cells were treated with BD Cytofix/Cytoperm (from BD Biosciences) solution for 20 min at 4°C. After two washing steps with 1x BD Perm/Wash solution, Ki67‐eFluor660 (20Raj1) was added to the cells for 30 min at 4°C. Finally, cells were washed twice in PBS plus 1% FCS and fixed in 1% paraformaldehyde for flow cytometry acquisitions on an LSRII‐Fortessa device (BD Biosciences). The data were analyzed with FlowJo software (TreeStar Inc.).

### 4.5. Differentiation Phenotyping

Cells were first stained with the LIVE/DEAD Fixable Aqua Dead Cell Stain Kit, for 15 min at room temperature, then washed then washed in PBS plus 1% FCS prior to staining with activation markers. Immunophenotyping was monitored using fluorochrome‐conjugated mAbs (from BD Biosciences, Beckman Coulter, Biolegend, ThermoFisher, or Miltenyi). We used the following antibodies: CD3‐eFluor450 (clone SK7), CD4‐AlexaFluor700 (OKT4), CD45RA‐PE‐Cy7 (5H9), CCR7‐PE‐Dazzle594 (G043H7), CD95‐PerCP Cy5.5 (DX2), and CD27‐APC (MT271). CD4+ T‐cells were incubated with the antibodies for 20 min at 4°C, then washed twice in PBS plus 1% FCS and fixed in 1% paraformaldehyde for flow cytometry acquisitions on an LSRII device (BD Biosciences). The data were analyzed with FlowJo software (TreeStar Inc.).

The different CD4+ T‐cell subsets were defined as follows: Naïve or TN: CD3+, CD4+, CD45RA+, CCR7+, CD27+, CD95‐; stem cell memory or TSCM: CD3+, CD4+, CD45RA+, CCR7+, CD27+, CD95+; central memory or TCM: CD3+, CD4+, CD45RA‐, CCR7+, CD27+; transitional memory or TTM: CD3+, CD4+, CD45RA‐, CCR7‐, CD27+; effector memory or TEM: CD3+, CD4+, CD45RA‐, CCR7‐, CD27‐; effector or TEMRA: CD3+, CD4+, CD45RA+, CCR7+/‐, CD27‐.

### 4.6. Quantitative RT‐PCR Arrays

After culturing, cells were counted, and a total of 20,000 cells per condition were placed in 96‐well plates containing VILO Reaction Mix, SUPERase‐In, and NP40 (all from ThermoFisher Scientific). RNA was denatured at 65°C for 90 s and then snap‐chilled on ice. Reverse transcription was performed with the 10× Superscript Enzyme Mix (Thermo Fisher Scientific) and T4 Gene 32 Protein (Merck), using the following cycling: 25°C: 5 min; 50°C: 30 min; 55°C: 25 min; 60°C: 5 min; and 70°C: 10 min. Specific target amplification (STA) was performed by adding TaqMan PreAmp Master Mix (ThermoFisher Scientific), 10× 96 primer mix, and 0.5 EDTA (Thermo Fisher Scientific) to complementary cDNA, using the following cycling: 96°C: 10 min; 20 cycles of 96°C: 5 s and 60°C: 4 min. The samples were then treated with exonuclease I (New England BioLabs) at 37°C: 30 min and 80°C: 15 min. All samples were 5‐fold diluted in 1× DNA suspension buffer (TEKnova). Exonuclease I‐treated, diluted samples were mixed with 2× SsoFast EvaGreen Supermix with low ROX (Bio Rad) and 20× DNA Binding Dye (Fluidigm), whereas the sets of Delta Gene primers (Fluidigm, Supporting Information 2: Table S1) were mixed with 2× Assay Loading Reagent (Fluidigm) and 1x DNA Suspension Buffer and then loaded on a primed 96.96 Dynamic Array chip (Fluidigm Cat: BMK‐M‐96.96). The chip was thermocycled in a Biomark (Fluidigm), and fluorescence was measured using the GE 96 × 96 PCR + Melt v.2 software. The baseline correction for the linear derivative mode was used. We used the web‐based comprehensive tool RefFinder to rank the stability of our housekeeping gene candidates [75]. Based on the expression stability in the investigated samples, IPO8 was determined as the best normalizing gene (Supporting Information 1: Figure S5), and the gene expression levels were plotted using 2^−ΔΔCt^, where ΔΔCt = ΔCt_SAMPLE_−ΔCt_CONTROL_, and ΔCt = Ct_TARGET GENE_−Ct_IPO8_.

### 4.7. Measurements of Cellular Metabolism

After culture, living cells were counted with an automatic Countess cell counter (Invitrogen) based on size and nonstaining with trypan blue. The number of living cells was then normalized before the analysis. Briefly, cells (2.5 × 10^5^) were resuspended in Seahorse XF RPMI medium, supplemented with sodium pyruvate (1 mM) and L‐glutamine (2 mM) in the presence or absence of glucose (10 mM), and added to Cell Tak‐coated wells (22.4 μg/mL, Corning). Cells were incubated for 50 min in a CO_2_‐free incubator at 37°C before loading the plate in the Seahorse analyzer. Different programs were run on the Seahorse analyzer depending on the assay. Mitochondrial function was analyzed by performing OCR and PER measurements before a subsequent three measurements following each compound injection. Drug Panel A: (1) XF RPMI medium, (2) oligomycin (2.5 μM), (3) FCCP (0.9 μM), and (4) rotenoneand antimycin A (1 μM) were injected through ports A, B, C, and D, respectively, for the mitochondrial stress test. Drug panel B: (1) XF RPMI media, (2) rotenone and antimycin A (0.5 μM), and (3) 2‐DG (50 mM), were injected for the glycolytic rate assay.

Basal OCR and PER are the mean values of the three baseline measures from three replicates (a mean of nine values in total). Maximal OCR and PER capacities were calculated as the mean value of three corresponding measurements from three replicates following injections of FCCP, oligomycin, or rotenone/antimycin, respectively.

### 4.8. Changes in Metabolic Genes Expression in Murine CD4+ T‐Cells

Normalized FC RNA‐seq data for murine CD4+ naïve T cells stimulated with IL‐2, IL‐7, or IL‐21 (each normalized to PBS) were provided by Christophe Benoist (Harvard Medical School), originally published in Baysoy et al. [35], in Nature Immunology (2023). To isolate metabolism‐related genes, we cross‐referenced all gene annotations with the KEGG Pathway Database to ensure comprehensive coverage of metabolic categories, including energy, amino acid, and lipid metabolism. Additionally, all genes beginning with the prefix “SLC,” representing solute carrier family nutrient transporters, were retained due to their established role in metabolic regulation. This resulted in a total of 1351 metabolic genes. To focus on cytokine‐specific transcriptional regulation and reduce confounding effects from commonly shared responses, we filtered this list to include only genes showing a FC greater than 1.5 or less than 0.67 in at least one of the three cytokine conditions (IL‐2, IL‐7, or IL‐21) (Supporting Information 3: Table S2). FC values were not log transformed. Pathway expression exploratory analyses were done with Reactome (explorer v3.7, database release #92) [76]. Heatmaps were generated in R (version 4.3.2) using the heatmaply package, with hierarchical clustering applied (Euclidean distance, complete linkage) and dendrograms hidden for clarity. A diverging color scale was applied using custom breaks and a nonlinear gradient: values <0.67 were shown in shades of blue (downregulation), values >1.5 in orange‐to‐red hues (upregulation), and values near FC = 1 in white, providing a more intuitive and biologically meaningful visual distinction between expression levels. FC values greater than 3 were capped at 3.

### 4.9. Statistical Analyses

Data were analyzed using GraphPad Prism software, version 9 (GraphPad Prism, La Jolla, CA). Data were presented as medians and ranges. Nonparametric one‐way analysis of variance (ANOVA) was used to assess the changes observed in the different cytokine conditions relative to the nonstimulated controls. Post hoc analyses were done using the Benjamini, Krieger, and Yekutieli method to correct for multiple comparisons (corrected *p* values are shown). The ratio‐paired *t*‐test was used for the comparison of paired relative data in two experimental conditions. *p* values of less than 0.05 were considered statistically significant. Heatmap and PCA analyses were done using Qlucore Omics Explorer v3.8, setting a *p* value <0.5 for multigroup comparisons and eliminating the sample ID as a confounding factor.

## Funding

Amal Elfidha was supported by the RHIVIERA Program through ANRS and MSDAVENIR funds. The study was supported with funds from the Institut Pasteur through the GPF LINMEC Program and a grant from the NIH‐National Institute of Diabetes and Digestive and Kidney Diseases (Grant R01DK131476).

## Conflicts of Interest

The authors declare no conflicts of interest.

## Supporting Information

Additional supporting information can be found online in the Supporting Information section.

## Supporting information


**Supporting Information 1** Figure S1: Impact of IL‐2, IL‐7, IL‐10, IL‐15, IL‐16, and IFN‐α on the size, granularity, CD3, CD4, and CD27 surface markers of CD4+ T‐cells. Figure S2: Gating strategy for CD4+ T‐cell subset distribution and the corresponding frequencies of each population. Figure S3: Expression of cytokine receptors in nonstimulated CD4+ T‐cells. Figure S4: Heatmap displaying differentially expressed genes in murine naïve CD4+ T‐cells. Figure S5: Ranking of housekeeping gene candidates for qPCR array analysis.

**Supporting Information 2** Table S1: Genes studied in transcriptomic qPCR analysis of human CD4+ T‐cells.

**Supporting Information 3** Table S2: Cytokine‐regulated metabolic genes in murine CD4+ naïve T‐cells.

## Data Availability

The data that support the findings of this study are available in the supporting information of this article.
