# Supplementary material for: Differential Effect of Cytokines on CD4+ T‐Cell Activation, Metabolism, and Susceptibility to HIV‐1 Infection
Source: J Immunol Res. 2026 Sep 2;2026:7633359. doi: 10.1155/jimr/7633359 (PMC13539073; doi:10.1155/jimr/7633359)
Supplement: Supplementary file 1 [file JIMR-2026-7633359-s002.pdf]

Supplementary material Legends

Table S1. Genes studied in transcriptomic q-PCR analysis of human CD4+ T cells

Table S2. Cytokine-regulated metabolic genes in murine CD4$^+$ naïve T cells. Metabolic genes regulated by IL-2, IL-7, or IL-21 in murine CD4$^+$ naïve T cells, based on transcriptomic data from Baysoy *et al.* (Baysoy *et al.*, 2023). Fold change values are normalized to PBS-treated controls. Only genes showing a fold change ≥1.5 (upregulated) or <0.67 (downregulated) in at least one cytokine condition were included. Fold change values were capped at 3 for heatmap visualization purposes.

Figure S1. Impact of IL-2, IL-7, IL-10, IL-15, IL-16 and IFN-α on the size, granularity, CD3, CD4 and CD27 surface markers of CD4+ T cells. Relative changes after 5 days of culture in presence of the cytokines when compared to the control condition. Each dot corresponds to one sample. Lines are medians.

Figure S2. Gating strategy for CD4+ T cell subset distribution and the corresponding frequencies of each population. Reported values are the fold-change in the contribution of each subpopulation to the total pool of CD4+ T cells for each culture condition compared to the non-stimulated condition. Each dot corresponds to one sample. Bars are medians.

Figure S3. Expression of cytokine receptors in non-stimulated CD4+ T cells. Proportion of CD4+ T cells expressing CD25 (IL-2Ra), CD122 (IL-2Rb), CD127 (IL-7Ra), CD215 (IL-15Ra). Each dot corresponds to one sample. Lines are medians.

Figure S4. Heat-map displaying genes that were differently regulated (FC ≥ 1.5 or <0.67) in CD4 naïve murine cells after stimulation with IL-2, IL-7, and IL-21 vs PBS-treated condition. Each row represents mean results for CD4+ T cells from 3 individual mice. Corrected FDR of Reactome metabolism related pathways for metabolic genes upregulated (FC>1.5, red) or

downregulated (FC<0.67, blue) upon stimulation of naïve murine CD4+ T cells with IL-7, IL-2 or IL-21. FDR>0.1 are not shown.

Figure S5. Ranking of housekeeping gene candidates after analysis with the RefFinder algorithm (Xie *et al.*, 2012). IPO8 was the most stable gene present in our panel and was used to normalize our qPCR data.

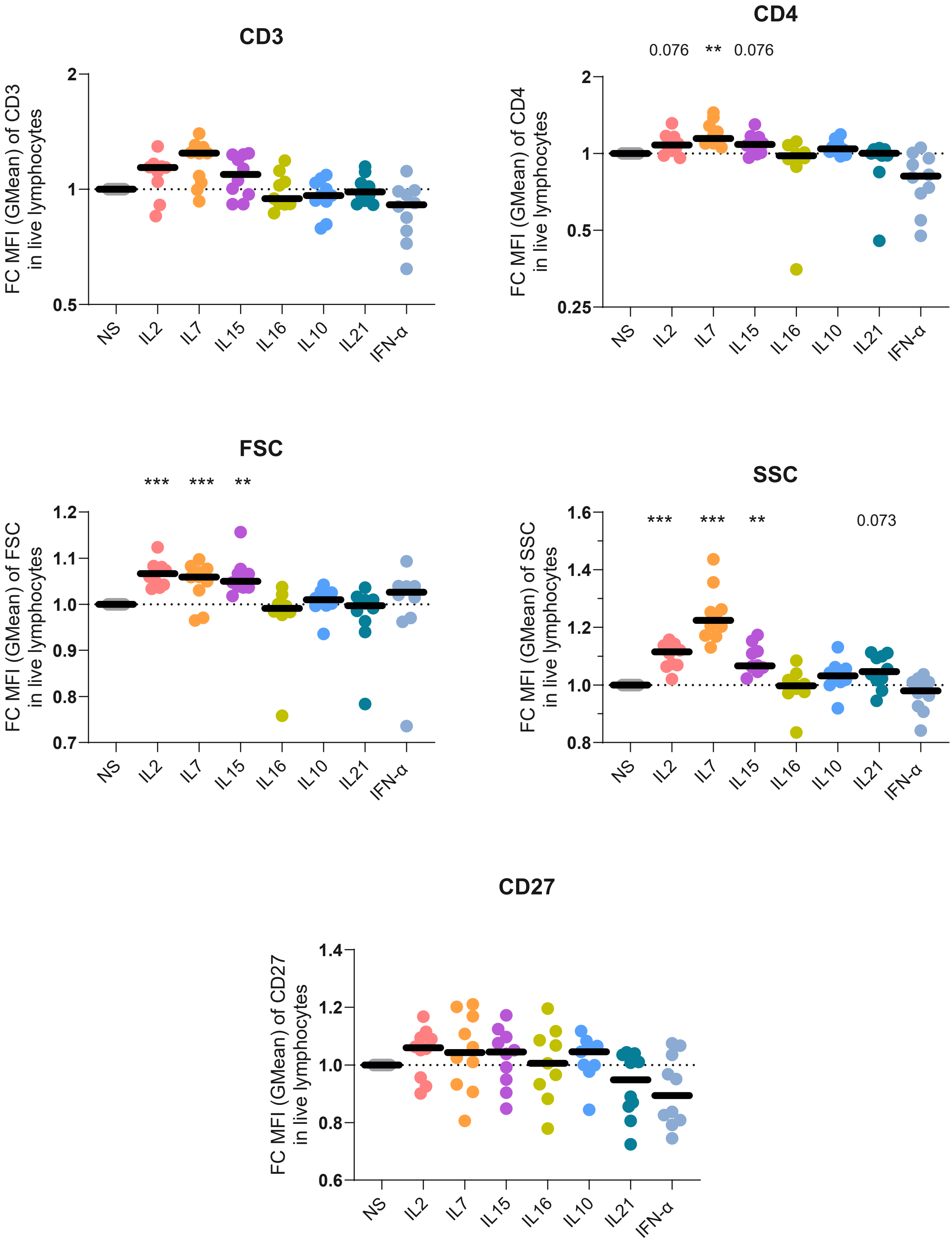
CD3
FC MFI (GMean) of CD3 in live lymphocytes
2
1
0.5
NS
IL2
IL7
IL15
IL16
IL10
IL21
IFN-α
CD4
0.076 ** 0.076
FC MFI (GMean) of CD4 in live lymphocytes
2
1
0.5
0.25
NS
IL2
IL7
IL15
IL16
IL10
IL21
IFN-α
FSC
*** *** **
FC MFI (GMean) of FSC in live lymphocytes
1.2
1.1
1.0
0.9
0.8
0.7
NS
IL2
IL7
IL15
IL16
IL10
IL21
IFN-α
SSC
*** *** ** 0.073
FC MFI (GMean) of SSC in live lymphocytes
1.6
1.4
1.2
1.0
0.8
NS
IL2
IL7
IL15
IL16
IL10
IL21
IFN-α
CD27
FC MFI (GMean) of CD27 in live lymphocytes
1.4
1.2
1.0
0.8
0.6
NS
IL2
IL7
IL15
IL16
IL10
IL21
IFN-α

Figure S1

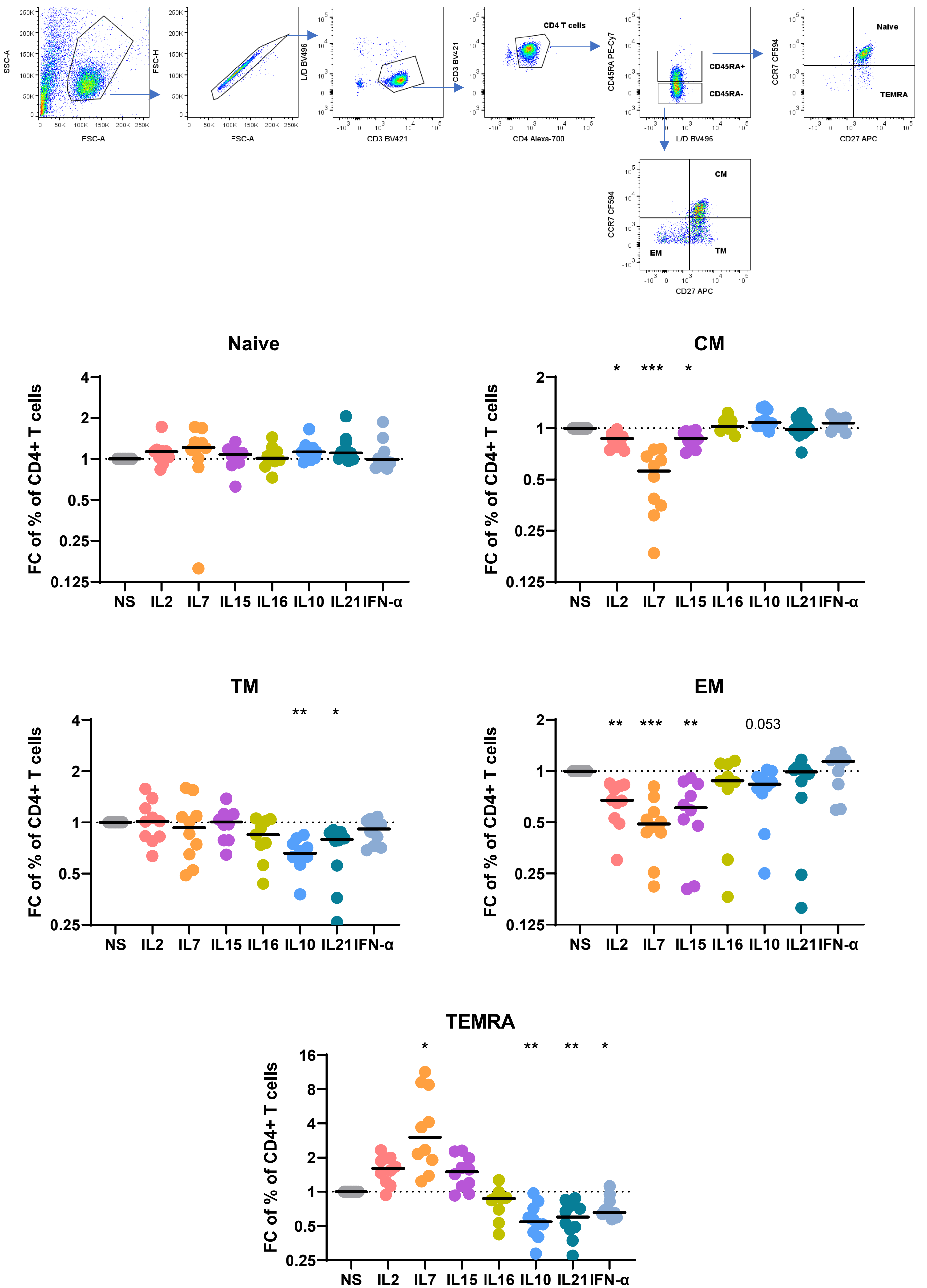

SSC-A
FSC-A
FSC-H
L/D BV496
CD3 BV421
CD4 T cells
CD4 Alexa-700
CD45RA PE-Cy7
CD45RA+
CD45RA-
CCR7 CF594
CD27 APC
Naive
TEMRA
CM
EM
TM
Naive
CM
TM
EM
TEMRA
FC of % of CD4+ T cells
NS IL2 IL7 IL15 IL16 IL10 IL21 IFN-α
0.053

Figure S2

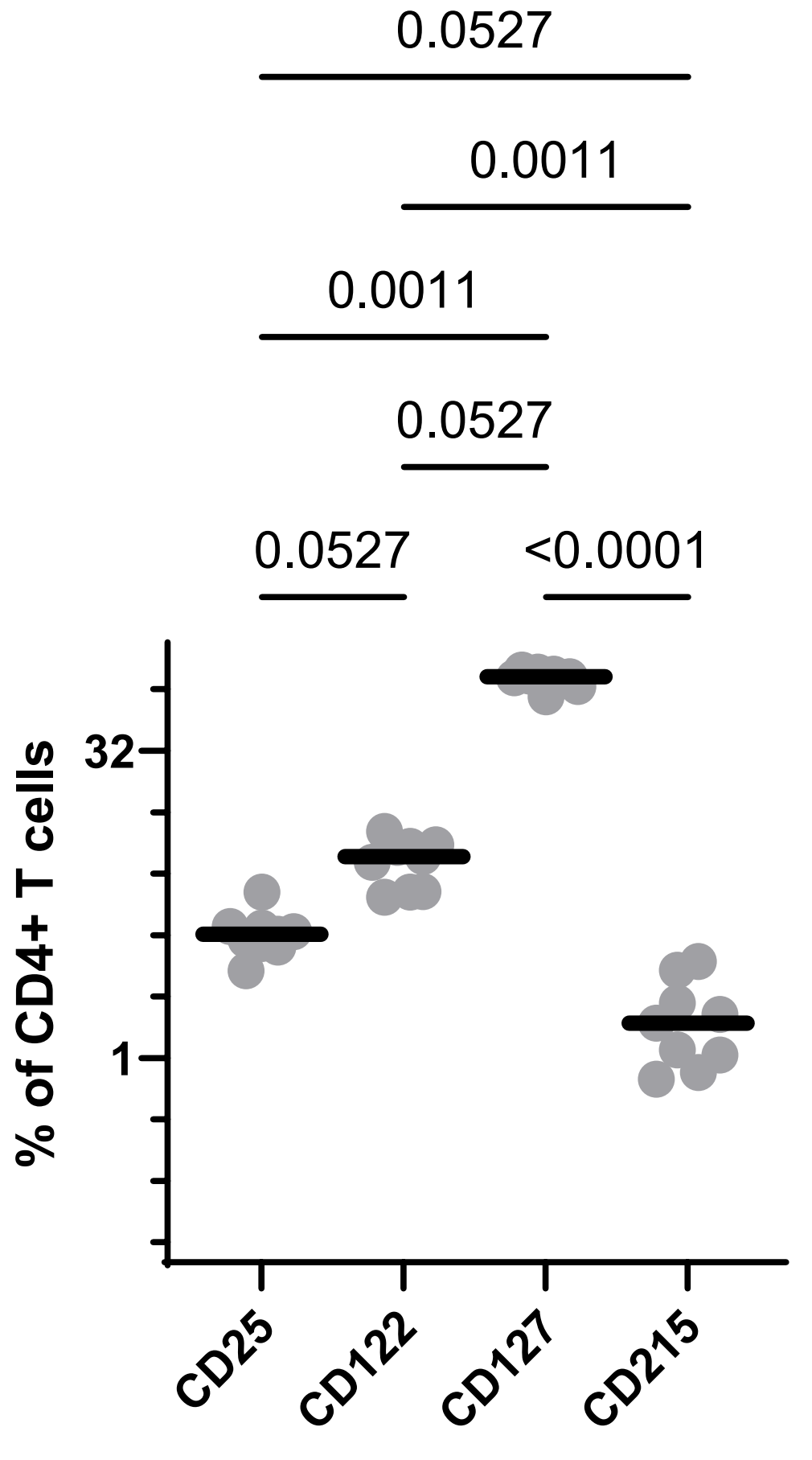
0.0527
0.0011
0.0011
0.0527
0.0527
<0.0001
32
1
% of CD4+ T cells
CD25
CD122
CD127
CD215

Figure S3

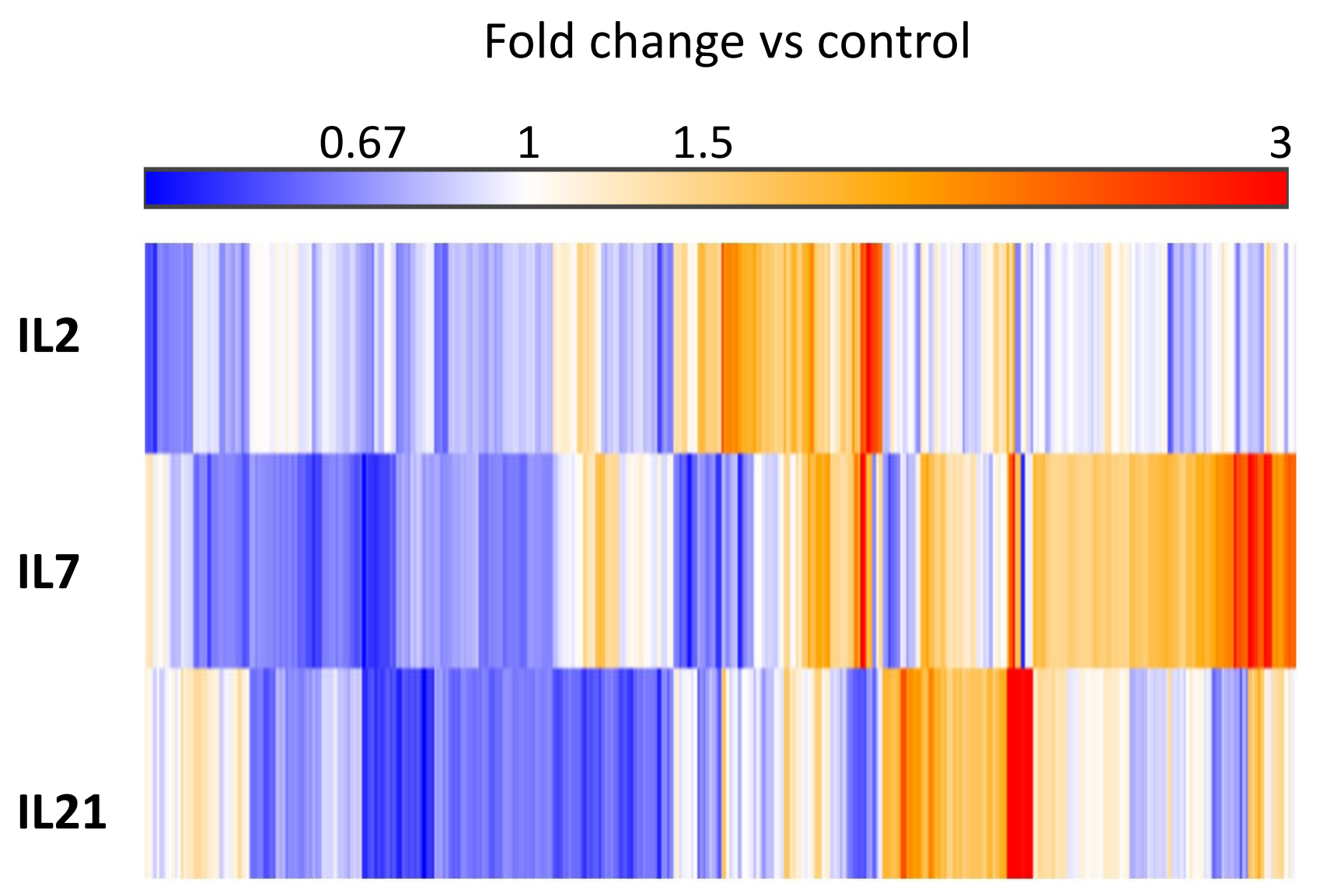

	IL7		IL2		IL21	
	**FC≥1.5**	**FC<0.67**	**FC≥1.5**	**FC<0.67**	**FC≥1.5**	**FC<0.67**
Metabolism	5.30E-14	8.77E-14	1.24E-09	1.57E-09	4.98E-07	9.03E-14
Metabolism of carbohydrates and carbohydrate derivatives	4.04E-03	7.80E-09	5.80E-03	1.99E-03		3.36E-06
Inositol phosphate metabolism						
Metabolism of lipids	6.36E-04			5.02E-05	5.21E-03	1.58E-03
Integration of energy metabolism	4.04E-03			3.97E-02	6.90E-02	
Metabolism of nitric oxide						
Aerobic respiration and respiratory electron transport						
Metabolism of nucleotides						
Metabolism of vitamins and cofactors	4.04E-03	4.40E-02	4.89E-02	8.60E-02		
metabolism of amino acids	9.75E-03					
metabolism of porphyrins						
biological oxidations				2.25E-02		

Figure S4

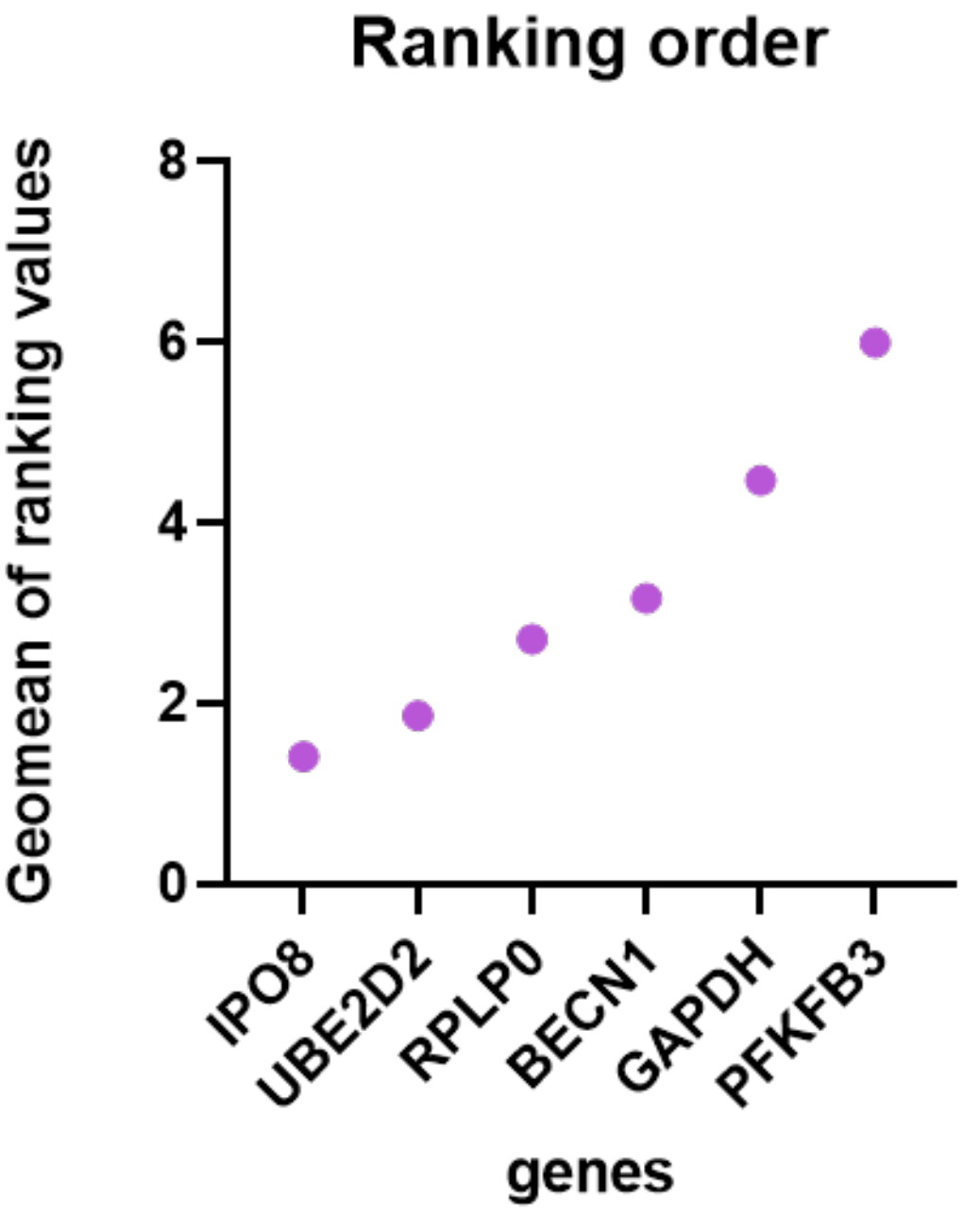
Ranking order
Geomean of ranking values
8
6
4
2
0
IPO8
UBE2D2
RPLP0
BECN1
GAPDH
PFKFB3
genes

Figure S5
